# P-1968. Pause Before You Prescribe and Let the Microbiome Thrive: A Health-System's Experience with a Best Practice Advisory for Antimicrobial Prescribing in Patients who Underwent Fecal Microbiota Transplant

**DOI:** 10.1093/ofid/ofaf695.2135

**Published:** 2026-01-11

**Authors:** Hunter O Rondeau, Samantha Linville, Sumaya Ased, Lawrence Elfman

**Affiliations:** SSM Health, Saint Louis, Missouri; SSM Health DePaul Hospital St. Louis, Bridgeton, Missouri; SSM Health, Saint Louis, Missouri; SSM Health, Saint Louis, Missouri

## Abstract

**Background:**

Fecal microbiota spores, live-brpk, an oral microbiota-based therapeutic, prevents recurrent Clostridioides difficile infection post-fecal microbiota transplant (FMT). Antibiotic exposure post-FMT can disrupt the restored microbiome. Following our health system’s formulary review of novel-FMT treatments, an OurPractice Advisory (OPA) was implemented to alert providers ordering antibiotics that the patient has previously received FMT. The desired action is to remove the antibiotic order when clinically appropriate or to replace it with an antibiotic of lower C.diff risk. This study evaluates the alert frequency and clinical effectiveness of the alert.

**Figure:**
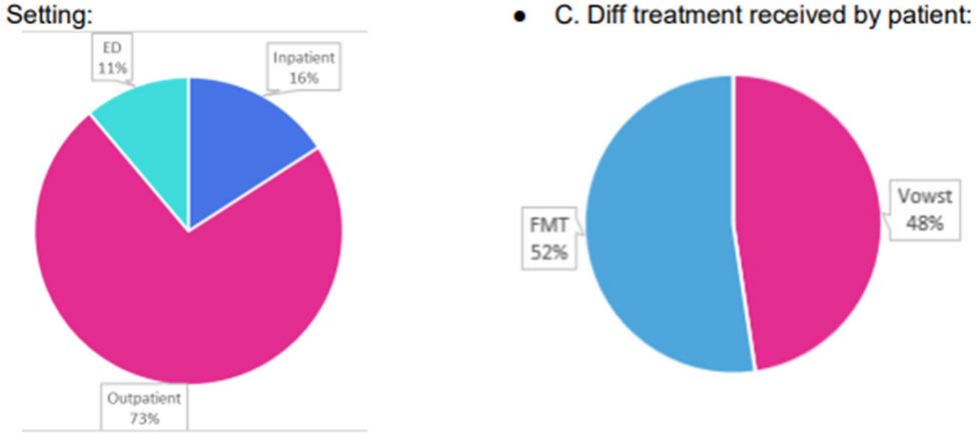
Setting and Type of FMT

**Figure:**
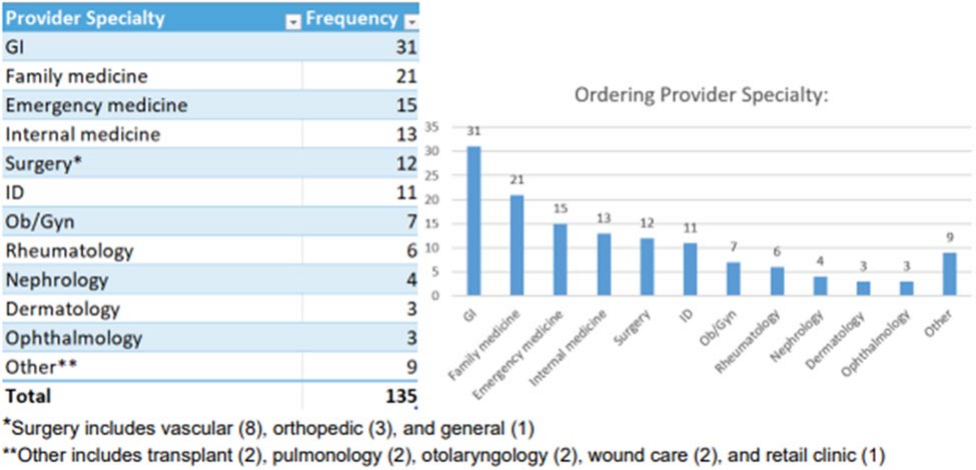
Ordering Provider Specialty

**Methods:**

A retrospective analysis was conducted on BPA alerts triggered for patients with prior fecal microbiota transplant who were prescribed antibiotics from July 1st 2024 through March 1st 2025. The study analyzed provider response patterns and actions taken upon alert activation to determine if the alert led to changes in therapy or achieved predefined success measures. This abstract was created with the assistance of Perplexity.

**Figure:**
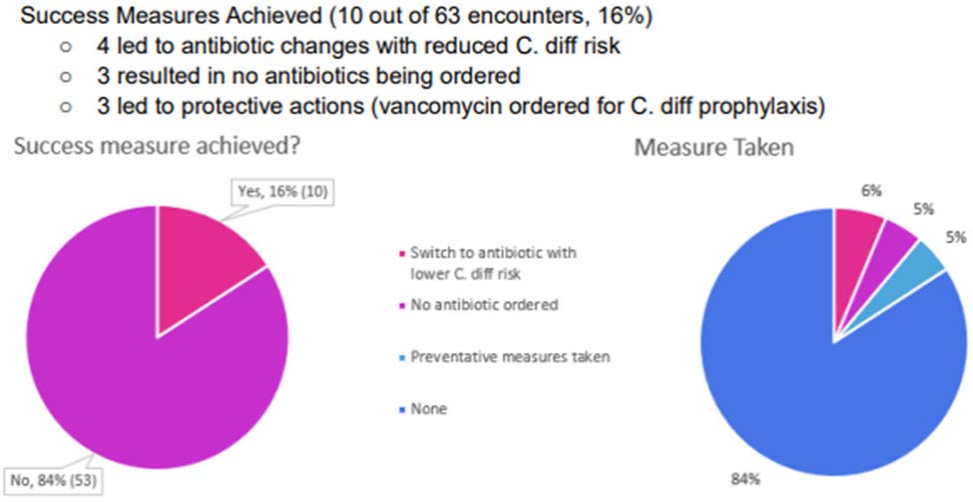
Outcomes

**Results:**

135 OPAs were triggered across 30 patients and 63 patient encounters [median 5.5 encounters per patient (IQR 2-7); median 2 alerts per encounter (IQR 1-3)]. The alerts spanned multiple specialties, including gastroenterology (31, 23%), family medicine (21, 16%), and emergency medicine (15, 11%). Success measures were achieved in 10 out of 63 encounters (16%), leading to antibiotic changes with less C. diff risk (4), no antibiotics ordered (3), or protective actions (3). Numerous alerts were acknowledged and overridden (71, 53%), resulting in no change. alerts were acknowledged and overridden (71, 53%), resulting in no change.

**Conclusion:**

The low success rate suggests a need to refine the BPA’s criteria, improve clinical relevance, and explore targeted provider education. Redesigning the alert to reduce fatigue and enhance actionability could improve outcomes for post-fecal microbiota spores, live-brpk patients. Future iterations should prioritize alert customization based on infection type and antibiotic spectrum to FMT.

**Disclosures:**

Hunter O. Rondeau, PharmD, BCIDP, ASHP: Honoraria|Cosmas Health: Advisor/Consultant|IDStewardship.com: Advisor/Consultant|Vituity: Honoraria

